# Supplementary material for: MAP2 is differentially phosphorylated in schizophrenia, altering its function
Source: Mol Psychiatry. 2021 Feb 1;26(9):5371–88. doi: 10.1038/s41380-021-01034-z (PMC8325721; doi:10.1038/s41380-021-01034-z)
Supplement: Supplementary file 7 — Table S6 [file 41380_2021_1034_MOESM7_ESM.pdf]

Table S6. Primer sequences

TARGET	FORWARD (5'-3')	REVERSE (5'-3')
S1782E mutagenesis	CCACGCTGGATCTGCCTGGTTCCTGT GTAATGATCTCAGCC	N/A
MAP2c sanger sequencing	Primer 1: GCGGTAGGCGTGTACGGT<br>Primer 2: TTCACGCACACCAGGCACT<br>Primer3: TAGACCTAAGCCATGTGACAT	Primer 1: AGCAGTCCCCAAGTCAGT<br>Primer 2: AGTGCCTGGTGTGCGTGAA<br>Primer3: GAGAAGGAGGCAGATTAGCTG
